# Differences and Similarities in Lipid Composition, Nutritional Value, and Bioactive Potential of Four Edible *Chlorella vulgaris* Strains

**DOI:** 10.3390/foods12081625

**Published:** 2023-04-12

**Authors:** Tatiana Maurício, Daniela Couto, Diana Lopes, Tiago Conde, Rita Pais, Joana Batista, Tânia Melo, Marisa Pinho, Ana S. P. Moreira, Mafalda Trovão, Ana Barros, Helena Cardoso, Joana Silva, Pedro Domingues, M. Rosário Domingues

**Affiliations:** 1 Mass Spectrometry Centre, LAQV-REQUIMTE, Department of Chemistry, University of Aveiro, Santiago University Campus, 3810-193 Aveiro, Portugal; 2 CESAM—Centre for Environmental and Marine Studies, Department of Chemistry, University of Aveiro, Santiago University Campus, 3810-193 Aveiro, Portugal; 3 Department of Medical Sciences and Institute of Biomedicine—iBiMED, University of Aveiro, 3810-193 Aveiro, Portugal; 4 Allmicroalgae Natural Products S.A., R&D Department, Rua 25 de Abril, 2445-287 Pataias, Portugal

**Keywords:** *Chlorella vulgaris*, lipidomics, polar lipids, PUFAs, antioxidant activity, anti-inflammatory activity

## Abstract

The microalga *Chlorella vulgaris* is a popular food ingredient widely used in the industry, with an increasing market size and value. Currently, several edible strains of *C. vulgaris* with different organoleptic characteristics are commercialized to meet consumer needs. This study aimed to compare the fatty acid (FA) and lipid profile of four commercialized strains of *C. vulgaris* (C-Auto, C-Hetero, C-Honey, and C-White) using gas- and liquid-chromatography coupled to mass-spectrometry approaches, and to evaluate their antioxidant and anti-inflammatory properties. Results showed that C-Auto had a higher lipid content compared to the other strains and higher levels of omega-3 polyunsaturated FAs (PUFAs). However, the C-Hetero, C-Honey, and C-White strains had higher levels of omega-6 PUFAs. The lipidome signature was also different between strains, as C-Auto had a higher content of polar lipids esterified to omega-3 PUFAs, while C-White had a higher content of phospholipids with omega-6 PUFAs. C-Hetero and C-Honey showed a higher content of triacylglycerols. All extracts showed antioxidant and anti-inflammatory activity, highlighting C-Auto with greater potential. Overall, the four strains of *C. vulgaris* can be selectively chosen as a source of added-value lipids to be used as ingredients in food and nutraceutical applications for different market needs and nutritional requirements.

## 1. Introduction

Microalgae are emerging organic and green ingredients with a wide diversity of nutrients and bioactive compounds, such as vitamins, minerals, proteins, and lipids. The consumption of microalgae-based products is on the rise, largely due to their beneficial health properties. They are excellent allies for a healthy and sustainable diet and lifestyle. Among commercialized microalgae, *Chlorella vulgaris* has one of the highest market values, expected to reach USD 412.3 million by 2028, and an actual yield of 5000 tons of dry matter per year [1]. *Chlorella vulgaris* is widely used in the food and feed industries, but also in the cosmetic and pharmaceutical fields, among others, as reviewed elsewhere [2].

Currently, the production of microalgae biomass at large scale is considered one of the most promising strategies to meet the needs of the next generations for food, feed, and pharmaceutical products [3]. Several cultivation methods have been established to produce *C. vulgaris,* leading to the development of different strains. The most common growth condition is autotrophic growth [4], which produces organic *C. vulgaris*, here called autotrophic *C. vulgaris* (abbreviated as C-Auto). A cost-effective alternative method to cultivate *C. vulgaris* is heterotrophic growth, resulting in heterotrophic *C. vulgaris* (C-Hetero) [3]. However, the organoleptic properties of C-Auto and C-Hetero, such as the strong colour, taste and odor, can limit its application in several products [5]. To overcome this constraint, two strains of *C. vulgaris* were developed from C-Hetero by selective breeding [6]. These strains, which are chlorophyll-deficient mutants, are characterized by a light white colour (abbreviated as C-White) and yellow colour (abbreviated as C-Honey) [6]. Compared to C-Auto and C-Hetero, the C-White and C-Honey strains exhibit a less intense odor and improved texture and taste, making them more attractive for applications in food products. Moreover, *C. vulgaris* strains (C-Auto, C-Hetero, C-White, and C-Honey) are recognized as safe and were approved for human consumption by the European Food Safety Authority (EFSA) and the Food and Drug Administration (FDA).

The different strains of *C. vulgaris* are distinguished biochemically by differences in the content in protein, carbohydrates, and pigments [6]. The C-Honey and C-White strains have a higher protein content when compared to C-Auto and C-Hetero, and lower carbohydrate and chlorophyll content. In addition, C-Honey and C-White have a remarkable presence of lutein and phytoene, respectively. However, in regard to the lipidome, little is known about the differences between these four *C. vulgaris* strains. Several studies have characterized the lipid profile of *C. vulgaris* grown autotrophically and heterotrophically (C-Auto and C-Hetero, respectively), describing a variety of complex lipids including glycolipids (GL) and phospholipids (PL), esterified with omega-3 and omega-6 polyunsaturated fatty acids (PUFAs) [7,8,9]. Omega-3 PUFAs are well-recognized for their beneficial effects on health status and are related to the prevention of non-communicable diseases (e.g., cardiovascular diseases, diabetes, and others) [10]. Complex lipids, such as PL, are described as excellent delivers of PUFAs, raising their bioavailability when compared to their free forms or esterified to triacylglycerols (TG) [11,12]. Furthermore, C-Auto and C-Hetero lipid extracts enriched in GL and PL bearing omega-3 and omega-6 PUFAs showed bioactive properties including antioxidant and anti-inflammatory effects [7,13]. Nevertheless, the lipidome of C-Honey and C-White strains has not been identified yet, and the differences between the lipid signatures of the previous *C. vulgaris* strains were never studied.

Thus, this work aims to compare the lipid profile and the bioactive properties of four edible *C. vulgaris* strains (C-Auto, C-Hetero, C-Honey, and C-White), to assess the predominant lipid differences between strains and their potential as functional ingredients for multiple applications.

## 2. Materials and Methods

### 2.1. Reagents

High-performance liquid chromatography (HPLC)-grade dichloromethane (CH_2_Cl_2_), absolute ethanol 96% (CH_3_CH_2_OH), and methanol (CH_3_OH) were obtained from Fisher Scientific Ltd. (Loughborough, UK). Milli-Q purified water (Synergy1, Millipore Corporation, Billerica, MA, USA) was used in all experiments. The 2,2-azino-bis (3-ethylbenzothiazoline-6-sulfonic acid) radical cation (ABTS^●+^) was purchased from Fluka (Buchs, Switzerland) and the α,α-diphenyl-β-pic-rylhydrazyl radical (DPPH^●^) from Aldrich (Milwaukee, WI, USA). All the remaining reagents were obtained from primary commercial sources. The lipid internal standards used in the lipidome analyses were acquired from Avanti Polar Lipids, Inc. (Alabaster, AL, USA): 1,2-dimyristoyl-*sn*-glycero-3-phosphate (dMPA), 1,2-dimyristoyl-*sn*-glycero-3-phospho-(10-rac-glycerol) (dMPG), 1,2-dimyristoyl-*sn*-glycero-3-phosphocholine (dMPC), 1,2-dipalmitoyl-*sn*-glycero-3-phosphatidylinositol (dPPI), 1,2-dimyristoyl-*sn*-glycero-3-phosphoethanolamine (dMPE), 1,2-dimyristoyl-*sn*-glycero-3-phosphatidylserine (dMPS), 1-nonadecanoyl-2-hydroxy-*sn*-glycero-3-phosphocholine (LPC), N-heptadecanoyl-D-*erythro*-sphingosine (Cer), and 1′,3′-bis-[1-2-di-tetradecanoyl-*sn*-glycero-3-phospho]-*sn*-glycerol (CL), and N-heptadecanoyl-D-*erythro*-sphingosylphosphorylcholine (SM).

### 2.2. Microalgae Strain and Culture Media

*Chlorella vulgaris* strains (C-Auto, C-Hetero, C-Honey, and C-White) were provided by Allmicroalgae. The C-Auto and C-Hetero *C. vulgaris* strains were produced as reported in previous works [3,7], whereas the chlorophyll-deficient mutants (C-Honey and C-White) were obtained by chemically induced random mutagenesis from C-Hetero, as described [6]. Briefly, the heterotrophic culture was grown in Guillard’s F2 culture medium adapted to local water using nitrate and glucose with a C:N ratio of 6.7:1. This culture was obtained sequentially from a 50–250 mL Erlenmeyer flask to a 5 L bench-top fermenter (New Brunswick BioFlo^®^ CelliGen^®^115; Eppendorf AG, Hamburg, Germany), and later to 200 L and 5000 L industrial fermenters. All fermenters were operated in fed-batch mode under controlled temperature, pH, and dissolved oxygen. *Chlorella vulgaris* cells were cultured and treated with different concentrations of ethyl methanesulfonate (EMS, Merck, New York, NY, USA) for 1 h in the dark. A colony of *C. vulgaris* mutants colored yellow (C-Honey) was selected by visual observation of the plates in dim light, and then isolated and sub-cultured several times on count agar plates (VWR, Carnaxide, Portugal). Then, C-Honey was grown to an exponential phase and subjected to a second round of random mutagenesis with 300 mM EMS. This time, a colony with a white colour (C-White) was selected and sub-cultured on count agar plates with 10 μM norflurazon (a metabolic inhibitor of carotenoids biosynthesis) and incubated at 30 °C in the dark for 1 week. C-White colonies were sub-cultured multiple times, with and without norflurazon to confirm mutant phenotypic stability. Mutant strains were considered stable after 50 generations expressing the same phenotype. To further ensure mutation stability, stock cultures of the mutants were maintained with the same inhibitor (10 µM norflurazon) to maintain the selective pressure used for the initial mutagenesis process and, thus, prevent reversion of the mutation.

### 2.3. Lipid Extraction

Total lipids were extracted according to the modified Folch’s method, as previously described [7,14]. The extraction was carried out individually for each *C. vulgaris* strain (C-Auto, C-Hetero, C-Honey, and C-White), from 25 mg of spray-dried biomass using a solvent mixture of CH_2_Cl_2_:CH_3_OH (2:1, *v*/*v*). All experimental procedures were carried out individually for five replicates (*n* = 5) obtained from a single large batch of biomass. The suspension was centrifuged at 2000 rpm for 10 min (Selecta JP Mixtasel, Abrera, Barcelona, Spain) and the supernatants were collected and dried under a stream of nitrogen. This step was repeated three times. Then, the dried extracts were re-extracted with 2 mL of CH_2_Cl_2_, 1 mL of CH_3_OH, and 0.75 mL of Milli-Q water, centrifuged (2000 rpm for 10 min), and then the lipid-containing layers were collected and dried. The aqueous phase was re-extracted with 2 mL of CH_2_Cl_2_ two more times. Finally, the extracts obtained were weighed and the extraction yield was calculated as a percentage of dry weight (*DW*), with the following Equation (1):

(1) $$Lipid\;yield\;\left(\%DW,\frac{w}{w}\right)=\frac{Weight\;of\;lipid\;extract\;\left(mg\right)}{Weight\;of\;biomass\;\left(mg\right)}\times 100$$

### 2.4. Fatty Acid Analysis by GC-MS

The fatty acid composition of *C. vulgaris* lipid extracts was analyzed by gas chromatography coupled to mass spectrometry (GC-MS). Fatty acid methyl esters (FAMEs) solution was prepared using alkaline trans-esterification of 60 μg of lipid extract and the addition of methyl nonadecanoate (internal standard) at 0.96 μg mL^−1^, as performed routinely in our laboratory [15,16]. The GC-MS was performed using an Agilent Technologies 8860 GC System (Santa Clara, CA, USA) interfaced with an Agilent 5977B Mass Selective Detector (Agilent, Santa Clara, CA, USA). The electron impact ionization was 70 eV, and full scan mode was used in 1 s cycles with a scanning range of *m/z* 50–550. A DB-FFAP capillary column (30 m long × 0.32 mm internal diameter, 0.25 µm film thickness, J&W Scientific, Folsom, CA, USA) was used. In the chromatographic analysis, the following conditions were used: injection volume of 2 μL (splitless), a constant flow rate of 1.4 mL min^−1^ of helium gas, inlet temperature 230 °C, and detector temperature 220 °C. The oven temperature was programmed at 58 °C for 2 min, 25 °C min^−1^ to 160 °C, 2 °C min^−1^ to 210 °C, 30 °C min^−1^ to 225 °C (held for 10 min). The data acquisition software was the GCMS5977B/Enhanced MassHunter. Agilent MassHunter Qualitative Analysis 10.0 software, NIST library, and the literature were used to identify FAs. The relative abundance (RA) of fatty acids was obtained by integrating the area under the peak and normalizing the data with the internal standard.

Finally, lipidic quality indices were determined. The atherogenic (AI), thrombogenic (TI) and hypocholesterolemic/hypercholesterolemic indexes (h/H) were calculated as proposed by Ulbricht and Southgate [17], with Equations (2)–(4), respectively. The peroxidation index (PoxI) was also calculated [18], according to Equation (5).

(2) $$\mathrm{AI}=\frac{[C12:0+(4\times C14:0)+C16:0]}{[\sum \mathrm{MUFA}+\sum \left(n-6\right)+\sum \left(n-3)\right]},$$

(3) $$\mathrm{TI}=\frac{\left[C14:0+C16:0+C18:0\right]}{[(0.5\times \mathrm{MUFA})+(0.5\times \sum \left(n-6\right))+(3\times \sum \left(n-3\right))+(\frac{\sum \left(n-3\right)}{\sum \left(n-6\right)})]}\;,$$

(4) $$\left(h/H\right)=\frac{[\mathrm{cis}-C18:1+\sum \mathrm{PUFA}]}{\left[C12:0+C14:0+C16:0\right]}\;,$$

(5) $$\mathrm{PoxI}=\left(\%\;monoenoic\;acid\;\;0.025\right)+\left(\%\;dienoic\;acid\times 1\right)+\left(\%\;\mathrm{trienoic}\;\mathrm{acid}\times 2\right)+\left(\%\;\mathrm{tetraenoic}\;\mathrm{acid}\times 4\right)+\;\left(\%\;\mathrm{pentaenoic}\;\mathrm{acid}\times 6\right)+\left(\%\;\mathrm{hexaenoic}\;\mathrm{acid}\times 8\right),$$

### 2.5. C18- Liquid Chromatography–Mass Spectrometry (C18–LC–MS)

Lipid extracts from *C. vulgaris* strains were analyzed by reverse-phase liquid chromatography (RP-LC-MS) in a Dionex Ultimate 3000 (Thermo Fisher Scientific, Bremen, Germany) using an Ascentis^®^ Express 90 Å C18 column (Sigma-Aldrich^®^, 2.1 × 150 mm, 2.7 µm) coupled to a Q-Exactive^®^ hybrid quadrupole Orbitrap mass spectrometer (Thermo Fisher, Scientific, Bremen, Germany). The mobile phase A was composed of Milli-Q water/acetonitrile (ACN) (40/60%) with 10 mM ammonium formate and 0.1% formic acid, while mobile phase B was composed of isopropanol/ACN (90/10%) with 10 mM ammonium formate and 0.1% formic acid. The following gradient was applied: 32% B at 0 min, 45% B at 1.5 min, 52% B at 4 min, 58% B at 5 min, 66% B at 8 min, 70% B at 11 min, 85% B at 14 min, 97% B at 18 min, 97% B at 25 min, 32% B at 25.01 min, and 32% B at 33 min.

For this analysis, a volume of 5 µL of a mixture containing 40 µg of lipid extract from each sample was dissolved in 20 µL of dichloromethane, 72 µL of a solvent system consisting of 50% isopropanol/50% methanol, and 8 µL of a mixture of phospholipid standards (dMPC—0.04 µg, SM d18:1/17:0—0.04 µg, dMPE—0.04 µg, LPC—0.04 µg, dPPI—0.08 µg, CL(14:0)4—0.16 µg; dMPG—0.024 µg, Cer 17:0/d18:1—0.08 μg, dMPS—0.08 µg; dMPA—0.16 µg), which was loaded into the column at 50 °C and at a flow-rate of 260 µL min^−1^.

The mass spectrometer operated simultaneously in positive (ESI 3.0 kV) and negative (ESI −2.7 kV) modes. The capillary temperature was 320 °C and the sheath gas flow was 35 U. Acquisition of data was performed in full scan mode with a high resolution of 70,000 and automatic gain control (AGC) target of 3 × 10^6^, in an *m/z* range of 300–1600, 2 micro scans, and a maximum injection time (IT) of 100 ms. Tandem mass spectra (MS/MS) were obtained with a resolution of 17,500, AGC target of 1 × 10^5^, 1 micro scan, and a maximum IT of 100 ms. The cycles consisted of a full-scan mass spectrum and 10 data-dependent MS/MS scans, which were repeated continuously throughout the experiments with a dynamic exclusion of 30 s and an intensity threshold of 8 × 10^4^. The normalized collision energy (CE) ranged between 20, 24, and 28 eV in the negative mode and 25 and 30 eV in the positive mode. Data acquisition was performed using the Xcalibur data system (V3.3, Thermo Fisher Scientific, Bremen, Germany).

Lipid species were identified using mass spectrometry-data independent analysis (MS-DIAL) v4.70 software and manual data analysis [19,20], and integrated in the MZmine v2.53 software [21]. The areas of the peaks of each lipid species were normalized by calculating the ratio against the area of the selected internal lipid standard (with the closest retention time). The relative abundance of each lipid species was estimated by dividing the normalized peak areas of each lipid species by the sum of the total normalized peak areas.

### 2.6. DPPH^●^ and ABTS^●+^ Radicals Scavenging Assays

Lipid extracts from *C. vulgaris* strains were tested for their antioxidant potential using two colorimetric scavenging assays: α,α-diphenyl-β-pic-rylhydrazyl (DPPH^●^) and the radical cation 2,2-azino-bis-3-ethylbenzothiazoline-6-sulfonic acid (ABTS^●+^). Briefly, 150 μL of an ethanolic dilution of the lipid extracts (25, 125, 250, and 500 μg mL^−1^) were mixed with 150 μL of DPPH^●^ or ABTS^●+^ working solution in ethanol (abs~0.9). The samples were incubated for 120 min, and the absorbance was measured at 517 nm for DPPH^●^ and 734 nm for ABTS^●+^ every 5 min using a UV/vis spectrophotometer (Multiskan GO 1.00.38, Thermo Scientific, Hudson, NH, USA). Controls were prepared by replacing the radical solution with ethanol. To monitor the radical stability, solutions with the radical plus ethanol were prepared. All measurements were carried out in triplicate. The same procedure was applied to the Trolox standard solution (12.5, 62.5, 125, 250 μg mL^−1^ in ethanol). The antioxidant activity was obtained using Equation (6), and is expressed as the percentage of inhibition of the DPPH^●^ (or ABTS^●+^), as follows:

(6) $$\mathrm{Inhibition}\%=\frac{\left(\mathrm{AbsRadical}-\left(\mathrm{AbsSample}-\mathrm{AbsControl}\right)\right)}{\mathrm{AbsRadical}}\times 100,$$

Abbreviations: AbsRadical, absorbance of radical (DPPH^●^ or ABTS^●+^); AbsSample, absorbance of the sample with radical (DPPH^●^ or ABTS^●+^); AbsControl, absorbance of the sample with ethanol.

The antioxidant activity expressed in Trolox equivalents (TE) was calculated with Equation (7), in which IC20 (or IC50) values are the concentration of lipid extract per sample and of Trolox, which promotes a 20% (or 50%) reduction in the radicals DPPH^●^ or ABTS^●+^.

(7) $$\mathrm{TE}\;\left(µ\frac{\mathrm{mol}}{g}\right)=\frac{\mathrm{IC}\;20\;\mathrm{or}\;\mathrm{IC}\;50\;\mathrm{Trolox}\;\left(µ\frac{\mathrm{mol}}{g}\right)}{\mathrm{IC}\;20\;\mathrm{or}\;\mathrm{IC}\;50\;\mathrm{of}\;\mathrm{samples}\;\left(µ\frac{g}{\mathrm{mL}}\right)}\times 1000,$$

### 2.7. Anti-Inflammatory Activity

The anti-inflammatory activity of the lipid extracts from *C. vulgaris* strains was evaluated with the cyclooxygenase-2 (COX-2) inhibition assay. This assay was carried out using the commercial COX-2 inhibitory screening assay kit, a Cayman test kit-701080 (Cayman Chemical Company, Ann Arbor, MI, USA), and was performed according to the manufacturer instructions. Briefly, lipid extracts were dissolved in 100% DMSO to a final concentration of 20, 60, and 125 μg mL^−1^. The amount of prostaglandin F2α produced was quantified by spectrophotometry (415 nm, Multiskan GO 1.00.38, Thermo Scientific, Hudson, NH, USA) and processed with the software SkanIT version 3.2 (Thermo Scientific). The results were expressed as a percentage of inhibited COX-2.

### 2.8. Statistical Analysis

Statistical analysis was performed using R version 4.0.2 [22], in Rstudio version 1.3.1093 [23]. Lipidomic data were log-transformed. All data was tested for normality using the Shapiro–Wilk test and homoscedasticity using the Levene test. When these assumptions were satisfied, ANOVA followed by post-hoc Tukey tests were used. Non-parametric data were examined using the Mann–Whitney U test, followed by post-hoc Dunn test. The *p*-values were corrected for multiple testing using the BH Benjamin, Hochberg, and Yekutieli method (*q*-values). All univariate analyses were performed using the R package ‘rstatix’ [24]. The principal component analysis (PCA) and the ellipses were created using the R libraries FactoMineR [25] and factoextra [26]. Heatmaps and hierarchical cluster analysis (HCA) were created using the R package pheatmap using “Euclidean” as clustering distance and “ward.D” as the clustering method [27]. The heatmaps were constructed based on the 50 lipid species with the lowest *q*-values (*q* < 0.05).

## 3. Results

### 3.1. Lipid Content and Fatty Acid Profile of C. vulgaris Strains

**Figure 1:**
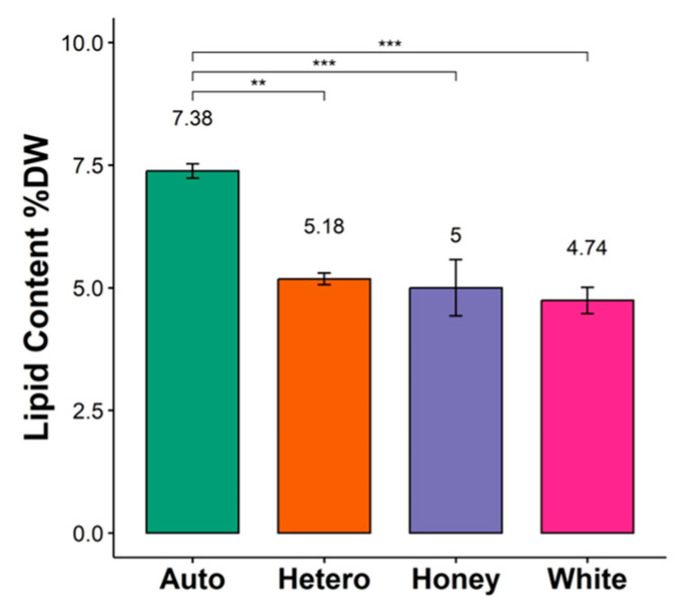
Lipid content of the four *C. vulgaris* strains (C-Auto, C-Hetero, C-Honey, and C-White) expressed in percentage % of biomass. Data represented as means ± standard deviation (SD), *n* = 5. ** q < 0.01, *** q < 0.001.

The lipid contents expressed in dry weight (DW) of C-Auto, C-Hetero, C-Honey, and C-White, estimated by gravimetry, were 7.38 ± 0.3%, 5.18 ± 0.2%, 5.0 ± 1.2%, and 4.74 ± 0.5%, respectively (Figure 1). Statistical analysis revealed that lipid content from C-Hetero, C-Honey, and C-White was significantly different from C-Auto. No significant differences were observed between the three heterotrophically grown strains (C-Hetero, C-Honey, and C-White).

The FA profile of C-Auto, C-Hetero, C-Honey, and C-White allowed the identification of 14 FAs (Table 1), including saturated (C14:0, C15:0, C16:0, C17:0, C18:0 and C20:0), monounsaturated (C16:1 and C18:1), and polyunsaturated (C16:2, C16:3, C18:2, C18:3) FAs. The relative abundances of each identified FAs were calculated, pinpointing the C18:3 (*n*−3) (α-linolenic acid, ALA) as the most abundant in C-Auto, while C16:0, C18:1 (*n*−9) (oleic acid, OA), and C18:2 (*n*−6) (linoleic acid, LA) were the most abundant in C-Hetero, C-Honey, and C-White, respectively. The total amount of omega-3 FAs was higher in C-Auto (41.1 ± 1.3), when compared to C-Hetero (10.3 ± 0.2), C-Honey (13.7 ± 0.5), and C-White (2.8 ± 0.3), while the total amount of omega-6 FAs was higher in C-Hetero (39.8 ± 0.9) and C-White (39.1 ± 2.3), followed by C-Honey (29.3 ± 0.5) and C-Auto (25.2 ± 0.6). The highest amount of MUFAs was verified in C-Honey (21.3 ± 0.3), followed by C-Hetero (19.7 ± 0.8), C-White (18.5 ± 1.0), and C-Auto (11.2 ± 0.4). The highest content of SFAs was detected in C-White (39.7 ± 3.4).

To estimate the lipid nutritional and health benefits of *C. vulgaris* strains, the lipid quality indexes including the *n*−6/*n*−3 ratio, atherogenic index (AI), thrombogenic index (TI), the hypocholesterolemic/hypercholesterolemic (h/H) index, and the peroxidation index (PI) were calculated (Table 2). C-White recorded the highest *n*−6/*n*−3 ratio (13.9 ± 0.6), with much lower ratios for the other three strains, with C-Hetero (3.9 ± 0.0), C-Honey (2.1 ± 0.0), and C-Auto (0.6 ± 0.0). The AI and TI are predictors of cardiovascular disease risk, which measure the probability of reducing atherogenic plaques and blood clot formation, respectively [28,29]. Lower values of AI and TI are associated with a higher protective effect against cardiovascular disease. The AI values (0.4 ± 0.0, 0.4 ± 0.0, and 0.5 ± 0.0) and TI (0.5 ± 0.0, 0.5 ± 0.0, and 1.1 ± 0.2) of C-Hetero, C-Honey, and C-White, respectively, were higher than for C-Auto (0.2 ± 0.0 for AI and TI). The h/H values were significantly lower in C-Hetero (2.8 ± 0.0), C-Honey (2.5 ± 0.0), and C-White (2.2 ± 0.1), when compared to C-Auto (4.7 ± 0.2). Higher values of h/H ratio are regarded as being beneficial for human health since they are correlated with a higher PUFA content [30]. In addition, the stability of FAs to peroxidation was assessed by calculating the PoxI [18]. C-Auto obtained the highest PoxI value, while the three heterotrophically grown strains (C-Hetero, C-Honey, and C-White) showed lower values. The higher the PoxI value, the more vulnerable the FAs to peroxidative damage.

### 3.2. Lipidomics Profile of Chlorella vulgaris Strains

The lipidome characterization of C-Auto, C-Hetero, C-Honey, and C-White allowed the identification of 316 lipid species present in 4 *C. vulgaris* strains distributed across polar lipids (GL, PL, and sphingolipids) and neutral lipids (NL). The GL classes identified were the digalactosyldiacylglycerol (DGDG) monogalactosyldiacylglycerol (MGDG) and sulfoquinovosildiacylglycerol (SQDG). The identified PL classes include phosphatidylcholine (PC), lysophosphatidylcholine (LPC), phosphatidylethanolamine (PE), phosphatidylglycerol (PG), and phosphatidylinositol (PI). Sphingolipids, including ceramides (Cer) and neutral lipids, diacylglycerols (DG) and TG, were also reported. The molecular compositions of the identified lipid species are described in Supplementary Table S1.

**Figure 2:**
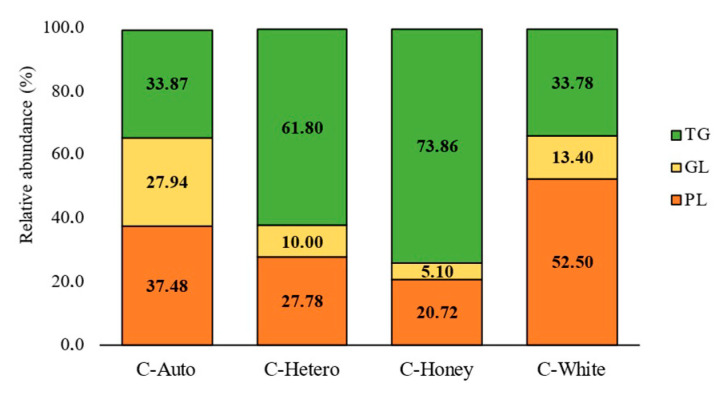
Graphical representation of the relative abundances (%), calculated by the sum of the normalized peak areas, of each main lipid group (phospholipids, glycolipids, and triacylglycerols) identified in the four *C. vulgaris* strains (C-Auto, C-Hetero, C-Honey, and C-White). GL—glycolipids; PL—phospholipids; TG—triacylglycerols.

The relative abundances of the main lipid groups identified (PL, GL, and TG) were calculated and are shown in Figure 2. Results show that C-Auto exhibits a balanced total relative abundance in PL, GL, and TG, while the three heterotrophically grown strains (C-Hetero, C-Honey, and C-White) show differences in the relative contents. The three heterotrophic strains have a lower content of GL compared to C-Auto. Moreover, C-Hetero and C-Honey have a higher relative abundance in TG, and C-White is characterized by a higher abundance in PL.

**Figure 3:**
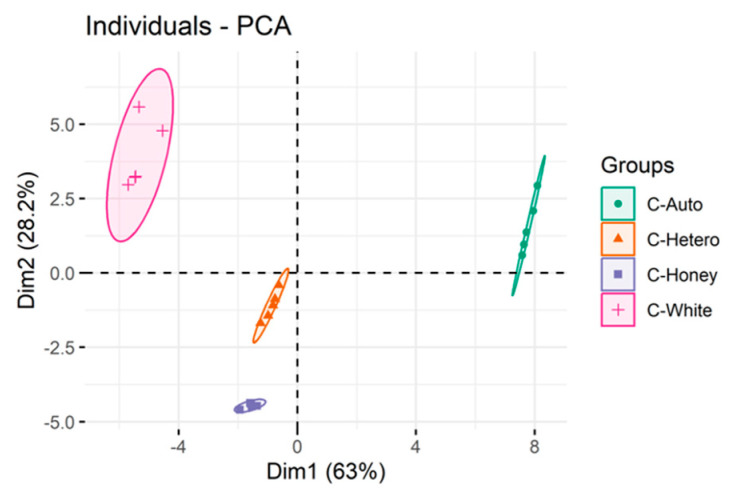
Principal component analysis scores plot of the 316 lipid species identified in all 4 *Chlorella vulgaris* strains (C−Auto, C−Hetero, C−Honey, and C−White).

Statistical analysis was carried out to compare the lipid profile of the four strains. Principal component analysis (PCA) of log-transformed levels of all individual lipid species identified (Figure 3) showed discrimination between the four strains, namely between C-Auto and the remaining three strains grown under heterotrophic conditions (C-Hetero, C-Honey, and C-White) along PC1 (63.0%). The strain C-White split from C-Hetero and C-Honey along PC2 (28.2%).

**Figure 4:**
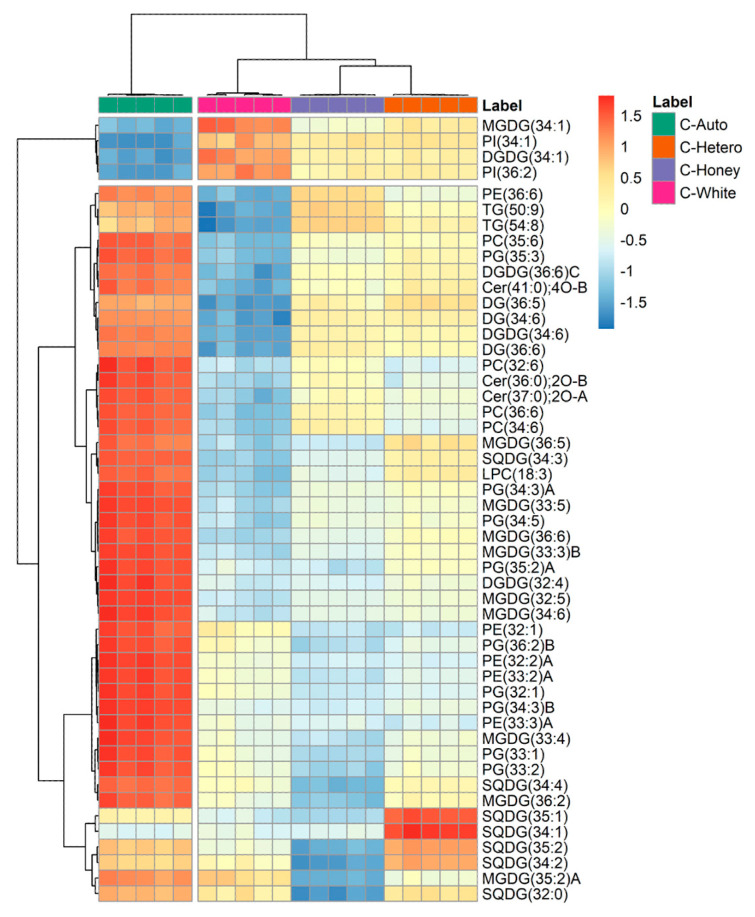
Two−dimensional hierarchical clustering analysis and heatmap containing the top 50 most discriminating lipids (one-way ANOVA, *q* < 0.05) identified in 4 strains of *Chlorella vulgaris* (C−Auto, C−Hetero, C−Honey, and C−White). Relative abundance levels are indicated on the colour scale, with numbers indicating the fold difference from the mean. The clustering of sample groups is represented by the dendrogram at the top. The clustering of individual lipid species considering their similarity in relative abundance is represented by the dendrogram on the left. Letters A, B, and C refer to different combinations of esterified fatty acids within the same molecular species, which can be found in Supplementary Table S1. Abbreviations: DGDG, digalactosyldiacylglycerol; MGDG, monogalactosyldiacylglycerol; SQDG, sulfoquinovosyldiacylglycerol; PC, phosphatidylcholine; LPC, lysophosphatidylcholine; PE, phosphatidylethanolamine; PG, phosphatidylglycerol; PI, phosphatidylinositol; Cer, ceramide; DG, diacylglycerols; TG, triacylglycerols.

Hierarchical clustering and heatmap analysis comprising a set of the top 50 most discriminant species with the lowest *q*-values (*q* < 0.05) between all 4 strains of *C. vulgaris* is represented in Figure 4. The hierarchical clustering showed that the C-Auto split from the other three strains on a first level, followed by a split between C-White and the remaining strains, in a similar trend as seen in PCA analysis. The 50 most discriminating lipids comprised lipids from all the identified classes. A total of 42 lipids (9 MGDG, 3 DGDG, 3 SQDG, 4 PC, 1 LPC, 5 PE, 9 PG, 2 TG, 3 DG, and 3 Cer) were up-regulated in C-Auto. The strain C-White had 1 MGDG, 1 DGDG, and 2 PI up-regulated compared to the remaining conditions, with the lowest content of these lipid species found in C-Auto. The remaining 46 species from the heatmap were downregulated or presented lower abundances in C-White. In C-Hetero, four SQDG were up-regulated when compared to the other strains, while the remaining discriminating lipid species were identified with lower abundance.

**Figure 5:**
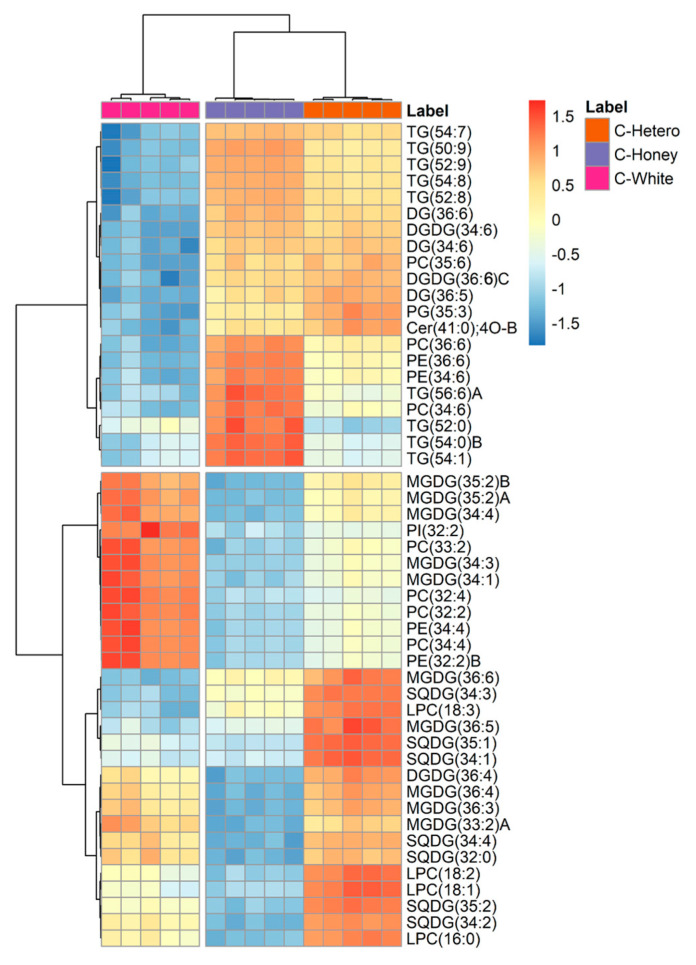
Two−dimensional hierarchical clustering analysis and heatmap containing the top 50 most discriminating lipids (One-Way ANOVA, *q* < 0.05) identified in 3 strains of *Chlorella vulgaris* (C−Hetero, C−Honey, and C−White). Relative abundance levels are indicated on the colour scale, with numbers indicating the fold difference from the mean. The clustering of sample groups is represented by the dendrogram at the top. The clustering of individual lipid species considering their similarity in relative abundance is represented by the dendrogram on the left. Letters A, B, and C refer to different combinations of esterified fatty acids within the same molecular species, which can be found in Supplementary Table S1. Abbreviations: DGDG, digalactosyldiacylglycerol; MGDG, monogalactosyldiacylglycerol; SQDG, sulfoquinovosyldiacylglycerol; PC, phosphatidylcholine; LPC, lysophosphatidylcholine; PE, phosphatidylethanolamine; PG, phosphatidylglycerol; PI, phosphatidylinositol; Cer, ceramide; DG, diacylglycerols; TG, triacylglycerols.

The level of discrimination between C-Auto and the other strains of *C. vulgaris* hinders comprehension of how the identified lipid species vary within C-Hetero, C-Honey, and C-White. To overcome this, hierarchical clustering and heatmap analysis comprising a set of the top 50 most discriminant species with the lowest *q*-values (*q* < 0.05) between C-Hetero, C-Honey, and C-White was performed (Figure 5). Results seem to indicate that C-Honey shows similarities with C-Hetero, while C-White exhibits a distinct lipid profile. A set of lipids comprising 13 lipid species (1 PC, 1 PG, 1 Cer, 2 DGDG, 3 DG, and 5 TG) had higher abundances in C-Hetero and C-Honey, but lower abundance in C-White. On the other hand, C-White was highlighted from C-Hetero and C-Honey, with a set of 12 lipids more abundant comprising GL (5 MGDG) and PL (1 PI, 2 PE, and 4 PC) esterified to LA. Overall, C-Honey showed a higher abundance of TG species with a low unsaturation degree, esterified to C18:1 and LA, while C-White showed the lowest abundance in TG species. In the case of C-Hetero, it discriminates from C-Honey, and C-White with 17 species of lipids, including mostly GL (1 DGDG, 5 MGDG, 7 SQDG) and 4 LPC.

### 3.3. Antioxidant Activity of Lipid Extracts from C. vulgaris Strains

**Figure 6:**
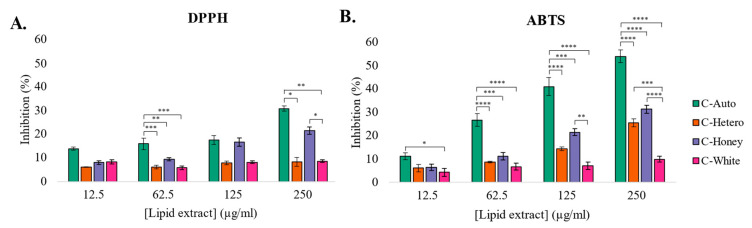
Antioxidant activity of C-Auto, C-Hetero, C-Honey, and C-White lipid extracts. Results expressed as percentage of inhibition induced by the concentration of lipid extract (μg mL^−1^): (**A**) DPPH^●^ radical, (**B**) ABTS^●+^ radical. Data represented as means ± SD, *n* = 3. * *q* < 0.05, ** *q* < 0.01, *** *q* < 0.001 and **** *q* < 0.0001.

The lipid extracts of the different *C. vulgaris* strains showed antioxidant activity, being able to distinctly inhibit the DPPH^•^ and ABTS^•+^ radicals in a dose-dependent manner (Figure 6).

The results obtained in the DPPH^•^ assay revealed that lipid extracts from C-Auto and C-Honey showed the highest antioxidant activity. C-Auto and C-Hetero extracts reached the inhibition of 20% of DPPH^•^ radicals corresponding to an IC20 of 108.2 ± 9.8 μg mL^−1^ with a TE of 93.1 ± 8.7 μmol Trolox g^−1^ for C-Auto and an IC20 of 228.9± 21.5 μg mL^−1^ and TE of 44.0 ± 4.2 μmol Trolox g^−1^ for C-Honey (Figure 6A). The lipid extracts from C-Hetero and C-White only promoted approximately 10% radical inhibition in all the concentrations evaluated. Statistically significant differences were observed at the 62.5 μg mL^−1^ of lipid extract between C-Auto and the three heterotrophically grown strains (C-Hetero, C-Honey, and C-White), as well as at the concentration 250 μg mL^−1^ between C-Auto and C-Hetero, C-Auto and C-White and C-Honey and C-White.

In the ABTS^•+^ assay, the lipid extracts from C-Auto showed the best antioxidant activity (Figure 6B). C-Auto promoted 50% of inhibition of the ABTS^•+^ radical, reaching an IC50 value of 230.7 ± 1.8 μg mL^−1^ and a TE activity of 854 ± 0.7 Trolox μmol g^−1^, while C-Honey and C-Hetero reached 20% inhibition of this radical, corresponding to an IC20 of 190.7 ± 13.4 μg mL^−1^ and TE of 42.1 ± 3.1 μmol Trolox g^−1^ for C-Hetero and an IC20 of 147.4 ± 10.6 μg mL^−1^ and a TE of 53.8 ± 4.0 μmol Trolox g^−1^ for C-Honey. C-White did not promote more than 10% radical inhibition in all concentrations evaluated. Statistically significant differences were observed in all concentrations of lipid extract tested (12.5, 62.5, 125, and 250 μg mL^−1^). Significant differences were observed between C-Auto and the three heterotrophically grown strains (C-Hetero, C-Honey, and C-White) at 62.5, 125, and 250 μg mL^−1^. Moreover, between the three heterotrophically grown strains, significant differences were seen at 125 μg mL^−1^ between C-Honey and C-White, and at 250 μg mL^−1^ between C-Hetero and C-White and C-Honey and C-White. These results suggest that *C. vulgaris* strains exhibit different antioxidant potential. Overall, lipid extracts from C-Auto had the best antioxidant activity in both scavenging assays (ABTS^●+^ and DPPH^●^), followed by C-Honey, C-Hetero, and, at last, C-White.

### 3.4. Anti-Inflammatory Activity of Lipid Extracts of C. vulgaris Strains

**Figure 7:**
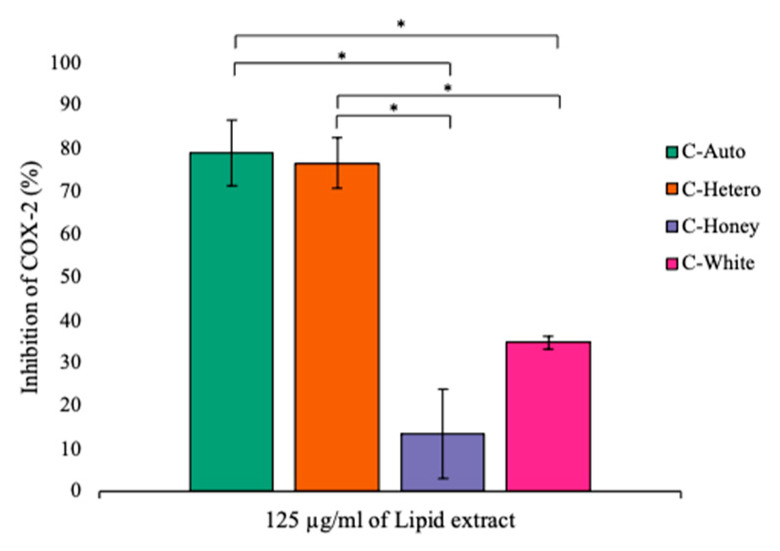
Anti-inflammatory potential (COX-2 assay) of lipid extracts from C-Auto, C-Hetero, C-Honey, and C-White. The results are expressed as a percentage of COX-2 inhibition as a function of the concentration of lipid extracts (125 μg mL^−1^). Data represented as means ± SD, *n* = 3. * *q* < 0.05.

The lipid extracts from all four *C. vulgaris* strains were able to inhibit COX-2 activity at a concentration of 125 μg mL^−1^ of lipid extract (Figure 7). However, at lower concentrations of 20 μg mL^−1^ of lipid extract, none of the *C. vulgaris* strains showed COX-2 inhibitory activity, and at 60 μg mL^−^, only C-Auto and C-Hetero lipid extracts demonstrated the ability to inhibit COX-2.

At the concentration of 125 μg mL^−1^ of lipid extract, C-Auto showed the highest inhibitory potential of COX-2 activity (79.1 ± 7.7%), followed by C-Hetero (76.8 ± 5.9%), C-White (34.9 ± 1.5%), and finally C-Honey (13.6 ± 10.5%). Statistical analysis revealed significant differences in the inhibitory COX-2 potential between (a) C-Auto and C-Honey and (b) C-Auto and C-White, as well as between (c) C-Hetero and C-Honey and (d) C-Hetero and C-White lipid extracts. These results suggest that lipid extracts from *C. vulgaris* strains, with different lipid composition, exhibited different anti-inflammatory potential.

## 4. Discussion

*Chlorella vulgaris* is one of the most commercialized microalga for food applications, with a high market value, and which has been widely used as food and as food ingredient [1,2]. Moreover, this microalga is rich in valuable nutrients, including lipids, such as PUFAs, which are described as having beneficial health effects. Most *C. vulgaris* biomass is produced under autotrophic conditions but other strains of *C. vulgaris* are also cultivated and commercialized, including strains obtained under heterotrophic conditions (herein called C-Hetero, C-Honey, and C-White), which reduces cultivation costs [3]. The strains C-Honey and C-White are also characterized by low pigment content and improved organoleptic properties, such as less flavor, smell, and colour [6]. Previous studies have shown that C-Auto and C-Hetero have different lipid profiles [7], while the lipidome of C-Honey and C-White strains were not identified before and were studied in this work for the first time. In addition, the bioactive potential of lipid extracts obtained from all *C. vulgaris* strains were evaluated and compared.

Between the four strains, C-Auto exhibited the highest lipid content (7.38 ± 0.3% in DW), while the remaining three strains showed similar content in lipids (approximately 5% in DW), but lower than C-Auto. These results are in agreement with previous reports on the lipid content of autotrophic *C. vulgaris* (approximately 9% in DW) [2,29] and the lower content of strains grown in heterotrophic conditions [7,9]. The dissimilar lipid accumulation between the four *C. vulgaris* strains may be due to cultivation conditions, such as light intensity, temperature, or CO_2_ supply [31,32]. The lower lipid content observed in the heterotrophically grown strains (C-Hetero, C-Honey, and C-White) can also be associated with the lower amount of chloroplasts in these strains, inferred by the lower glycolipid (lipids typical of chloroplasts) content and pigments, particularly chlorophylls, which are in lower abundance in these strains [3,6].

The FA profile analysis revealed that C-Auto showed a higher content of omega-3 PUFAs, with ALA representing the most abundant FA, while the lowest omega-3 content was reported for C-White. In contrast, C-Hetero and C-White exhibited the highest content in omega-6 PUFAs, in similar levels, whereas C-Auto showed the lowest omega-6 PUFAs amount. These results are in accordance with previous reports on the comparison of *C. vulgaris* grown auto- and heterotrophically [7,30]. The four C. vulgaris strains can be used as source of essential PUFAs, such as ALA and LA, which can only be obtained through diet and are important precursors of longer omega-3 and omega-6 PUFAs, respectively. The studied *C. vulgaris* strains have higher abundances in ALA and LA when compared to other microalgae, and other abundant sources of LA (*e.g.,* canola oil) [33]. Omega-3 PUFAs are often associated with health benefits in immunity regulation, while a moderate intake of omega-6 PUFAs is associated with lower risk of cardiovascular disease, highlighting the importance of having dietary sources of both omega-3 and omega-6 PUFAs in different abundances [10,34]. Despite the four *C. vulgaris* strains having a distinct FA profile, they exhibit an interesting nutritional composition in terms of FA composition. Therefore, they can represent a source of omega-3 and omega-6 PUFAs, providing health benefits for people with different nutrient deficiencies in these PUFAs. Another abundant FA in *C. vulgaris* strains produced heterotrophically (C-Hetero, C-Honey, and C-White) was the OA. This FA is quite abundant in olive oil and is described with a protective effect against inflammation and cancer [35,36].

According to the calculated index values, the four *C. vulgaris* strains had good lipidic nutritional indicators, including AI and TI, which are cardiovascular disease risk predictors, and h/H ratio (Table 2). Lower values of AI and TI usually indicate a higher protective effect against cardiovascular disease [30], and were observed in the four *C. vulgaris* strains. On the other hand, an elevated h/H is associated with a high content in terms of PUFAs, and a beneficial effect by lowering cholesterol levels [34,37]. The four *C. vulgaris* strains showed high values of h/H, and C-Auto excelled with the best ratio. These results support the nutraceutical value of FAs from the studied *C. vulgaris* strains. Indeed, the health potential of FAs from *C. vulgaris* were already addressed in the literature. For instance, in mice fed with a high-fat diet, the administration of FAs from *C. vulgaris* promoted a beneficial effect on hyperglycemia, hyperlipidemia, hepatic steatosis, and adipocyte hypertrophy [38]. Another study evaluated the effect of auto- and heterotrophic *C. vulgaris* in zebrafish and showed that both strains have anti-obesity, anti-steatosis, and anti-inflammatory activity [39]. The susceptibility of the lipid extracts of the four *C. vulgaris* strains was also assessed. Lower values of PoxI were observed for the heterotrophically grown strains (C-Hetero, C-Honey, and C-White), of which C-White achieved the best index value, suggesting a higher stability of the FAs present in the lipid extract [18,40].

The lipid profile of the four strains of *C. vulgaris* was determined by lipidomic analysis using high-resolution RP-LC-MS/MS, which allowed the identification of 316 lipid species, including polar and neutral lipids. The polar lipid species were similar in all four strains, and in accordance with data previously reported in the literature for C-Auto and C-Hetero analyzed by HILIC-LC-MS/MS [7,8]. However, differences in the relative amounts of the lipid species were found between the strains (Figure 4), suggesting a specific lipid signature for each strain but with similarities between the strains grown heterotrophically. For instance, C-Auto stands out, with a higher amount of lipids esterified to PUFAs, especially GL (MGDG and DGDG), while C-Hetero, C-Honey, and C-White were characterized by abundant lipids with a lower unsaturation degree. Comparing the three strains grown under heterotrophic conditions (C-Hetero, C-Honey, and C-White), changes in lipid composition were observed between them. Lipid extracts from C-Hetero and C-Honey strains exhibited a high abundance of TG species, including a wide diversity of TG molecules mainly esterified to OA and essential FAs, namely ALA and LA. The TGs are main components of edible oils, and the physiochemical and nutritional properties of these are determined by the TG molecules present [41]. Edible oils are rich in omega-3 and -6 PUFAs, and are currently obtained from fish (e.g., fish oil), which represents a limited supply, reinforcing the need for sustainable sources of edible oils [42]. Moreover, the production volume as well as the market value of fish oil is expected to increase, demanding fast and sustainable alternatives [43,44]. Therefore, microalgae including *C. vulgaris*, can be a promising source of edible oils and an alternative to fish oil for applications in food, feed, and in dietary supplements.

Polar lipids, such as GL, are highlighted as efficient delivers of PUFAs, with better bioavailability compared with TGs [12,45]. Polar lipids are the main components of lecithins, which are used as food ingredients and in the formulation of nutraceuticals [46]. In addition, a few polar lipids from microalgae have been reported as modulators of inflammation, such as some PC, PG, MGDG, DGDG, and SQDG lipids [29], which were all identified in the four *C. vulgaris* strains. Moreover, species, such as MGDG (32:0), MGDG (32:2), MGDG (34:1), and SQDG (32:0), identified in all *C. vulgaris* strains and with a higher relative abundance in C-White, were previously reported with anti-viral, anti-obesity, and anti-tumor activities [47,48,49,50]

The bioactive potential of *C. vulgaris* strains was assessed through DPPH^•^ and ABTS^•+^ radicals scavenging assays. All lipid extracts from *C. vulgaris* strains exhibited antioxidant activity, with C-Auto showing the best antioxidant potential and C-White the lowest (Figure 6). Lipid extracts from C-Hetero, C-Honey, and C-White have a similar content in the total amount of PUFAs, with different proportions in omega-3 and omega-6 PUFAs, and dissimilar antioxidant activities. The lower antioxidant activity of C-Hetero, C-Honey, and C-White extracts could be correlated with the *n*−6/*n*−3 (Table 2), and the lower abundance of omega-3 PUFAs when compared with C-Auto. However, differences among the antioxidant effect between the strains grown heterotrophically can be due to the different omega-3 and omega-6 PUFA contents, or the contribution of the synergistic effects of other compounds with antioxidant properties, such as pigments, cannot be ruled out [6]. Altogether, results suggest that *C. vulgaris* strains can be considered a promising source of antioxidant lipids for the formulation of added-value products for the food and nutraceutical industries. The anti-inflammatory potential of lipid extracts was also assessed by the in chemico COX-2 inhibitory assay. Lipid extracts from all four strains of *C. vulgaris* inhibited the activity of COX-2, with C-Auto and C-Hetero recording the highest anti-inflammatory activity. Lipid extracts from C-Auto and C-Hetero were already described with anti-inflammatory potential [7,39]. When compared to other microalgae (*Gloeothece* sp., *Tetraselmis* sp. and *Phaeodactylum tricornutum*) tested at similar and higher concentrations (500 μg mL^−1^) of the lipid extracts [51,52], C-Auto and C-Hetero (125 μg mL^−1^) revealed higher anti-inflammatory potential, while C-White showed similar activity. Differences in the anti-inflammatory potential observed between heterotrophically grown strains (C-Hetero, C-Honey, and C-White) can be associated with the lipid species competing as a substrate for COX-2. As previously mentioned, algal lipids have been reported as modulators of inflammation, and several PL and GL species may play an important role in reducing unregulated inflammatory processes [53].

The four *C. vulgaris* strains evaluated in this study seem to represent a source of bioactive lipids with functional properties, ones that can be applied in the formulation of promising new ingredients in the development of foods and dietary supplements. Moreover, they are an interesting alternative for consumers with different nutritional requirements or with plant-based diets, such as vegetarians and vegans.

## 5. Conclusions

This work described the differences in FA and lipid profiles, as well as the antioxidant and anti-inflammatory properties of four strains of *C. vulgaris* (C-Auto, C-Hetero, C-Honey, and C-White). These strains are already approved for human consumption and marketed as food ingredients and dietary supplements. The same FAs, lipid classes, and lipid species were found in the lipidome of all *C. vulgaris* strains. Despite the similarities, statistical analysis showed a clear difference in the relative abundance of certain FAs, lipid classes, and species, revealing a strain-specific lipid signature. The autotrophically grown *C. vulgaris* was highlighted with a high abundance in omega-3 PUFAs and GL esterified to PUFAs, while the heterotrophically grown strains showed a higher abundance of low unsaturated species. Moreover, C-Hetero and C-Honey were abundant in TG species, esterified to OA and LA, while C-White had a higher content in polar lipids. The four strains exhibited antioxidant properties, with the highest value verified in C-Auto. Furthermore, among the four strains, C-Auto and C-Hetero exhibited the best anti-inflammatory potential. Altogether, these results contribute to the valorization of all four strains of *C. vulgaris* as food ingredients and supplements with nutritional and functional value for different markets.

## Figures and Tables

**Table 1:** GC-MS analysis of the fatty acid composition (in %) of total lipid extract from the four *C. vulgaris* strains (C-Auto, C-Hetero, C-Honey, and C-White). Data are presented as mean ± SD (*n* = 5). The most abundant FAs are highlighted in bold. Values with the same letter (^a^, ^b^, or ^c^) are statistically different (*q* < 0.05).

Fatty Acid	C-Auto	C-Hetero	C-Honey	C-White
C14:0	0.2 ± 0.0 ^a,b^	0.2 ± 0.0 ^a^	0.2 ± 0.0	0.2 ± 0.0 ^b^
C15:0	0.4 ± 0.0 ^a,b,c^	0.1 ± 0.0 ^a^	0.1 ± 0.0 ^b^	0.1 ± 0.0 ^c^
**C16:0**	**15.6 ± 0.3 ^a,b^**	**23.7 ± 0.4 ^c^**	**24.7 ± 0.4 ^a^**	**26.3 ± 0.6 ^b,c^**
C16:1(*n*−9)	1.1 ± 0.0 ^a,b^	2.1 ± 0.0	2.7 ± 0.1 ^a^	2.8 ± 0.2 ^b^
C16:1(*n*−7)	2.9 ± 0.1 ^a^	0.2 ± 0.0	0.2 ± 0.0	0.1 ± 0.0 ^a^
C16:2(*n*−6)	**7.2 ± 0.2**	**7.1 ± 0.2**	3.8 ± 0.2 ^a^	8.6 ± 0.6 ^a^
C17:0	0.7 ± 0.0 ^a^	0.3 ± 0.0	0.8 ± 1.0	0.2 ± 0.0 ^a^
C16:3(*n*−3)	**14.8 ± 0.5 ^a,b^**	3.4 ± 0.1 ^a^	4.7 ± 0.3 ^c^	1.0 ± 0.1 ^b,c^
**C18:0**	5.5 ± 2.4 ^a^	5.6 ± 2.2 ^b^	**9.6 ± 0.4**	**12.7 ± 3.4 ^a,b^**
**C18:1(*n*−9)**	4.5 ± 0.4 ^a,b^	**16.6 ± 0.7 ^a^**	**18.0 ± 0.2 ^b,c^**	**15.3 ± 0.8 ^c^**
C18:1(*n*−7)	2.7 ± 0.1 ^a,b^	0.8 ± 0.1 ^c^	0.4 ± 0.0 ^b^	0.3 ± 0.1 ^a,c^
**C18:2(*n*−6)**	**18.0 ± 0.5 ^a,b^**	**32.7 ± 0.7 ^a,c^**	**25.5 ± 0.3 ^c^**	**30.5 ± 1.8 ^b^**
C18:3(*n*−3)	**26.3 ± 0.8 ^a,b^**	6.9 ± 0.1 ^a^	**9.0 ± 0.2 ^c^**	1.8 ± 0.1 ^b,c^
C20:0	0.1 ± 0.0 ^a^	0.2 ± 0.0 ^b^	0.4 ± 0.0 ^a,b^	0.2 ± 0.1
Σ SFA	22.5 ± 1.5 ^a,b,c^	30.1 ± 1.8 ^a,d^	35.8 ± 1.1 ^b^	39.7 ± 3.4 ^c,d^
Σ MUFA	11.2 ± 0.4 ^a,b^	19.7 ± 0.8 ^a^	21.3 ± 0.3 ^b,c^	18.5 ± 1.0 ^c^
Σ PUFA	66.3 ± 1.5 ^a,b^	50.1 ± 1.0	43.0 ± 0.9 ^a^	41.9 ± 2.5 ^b^
**Σ (*n*−3)**	41.1 ± 1.3 ^a,b^	10.3 ± 0.2 ^a^	13.7 ± 0.5 ^c^	2.8 ± 0.3 ^b,c^
**Σ (*n*−6)**	25.2 ± 0.6 ^a,b^	39.8 ± 0.9 ^a^	29.3 ± 0.5	39.1 ± 2.3 ^b^

Abbreviations: ∑ PUFA, the sum of polyunsaturated fatty acids; ∑ MUFA, the sum of monounsaturated fatty acids; ∑ SFA, the sum of saturated fatty acids.

**Table 2:** Lipidic quality indexes of the total lipid extracts of four *Chlorella vulgaris* strains, C-Auto, C-Hetero, C-Honey, and C-White. Data are represented as means ± SD, *n* = 5. Values with the same letter (^a^, ^b^, or ^c^) are statistically different (*q* < 0.05).

Indexes	C-Auto	C-Hetero	C-Honey	C-White
*n*−6/*n*−3	0.6 ± 0.0 ^a,b^	3.9 ± 0.0 ^a^	2.1 ± 0.0 ^c^	13.9 ± 0.6 ^b,c^
AI	0.2 ± 0.0 ^a,b^	0.4 ± 0.0 ^c^	0.4 ± 0.0 ^a^	0.5 ± 0.0 ^b,c^
TI	0.2 ± 0.0 ^a,b^	0.5 ± 0.0 ^c^	0.5 ± 0.0 ^a^	1.1 ± 0.2 ^b,c^
h/H	4.7 ± 0.2 ^a^	2.8 ± 0.0 ^b^	2.5 ± 0.0	2.2 ± 0.1 ^a,b^
PoxI	107.8 ± 2.7 ^a^	61.1 ± 1.2	58.9 ± 1.4	45.1 ± 2.7 ^a^

Abbreviations: AI, atherogenic index; TI, thrombogenic index; h/H ratio, hypocholesterolemic/hypercholesterolemic index; PoxI, peroxidation index.

## Data Availability

The datasets generated during and/or analyzed during the current study are available from the corresponding authors on reasonable request.

## Supplementary Materials

The following supporting information can be downloaded at: https://www.mdpi.com/article/10.3390/foods12081625/s1, Table S1: Exact mass measurements of all the lipid species identified by RP-LC-MS in lipid extracts of four *C. vulgaris* strains (C-Auto, C-Hetero, C-Honey, and C-White). C represents the total number of carbon atoms and N the total number of double bonds on the fatty acyl chains. Letters A, B, and C refer to different combinations of esterified fatty acids within the same molecular species.
